# Exploring the relationship between life quality and the perceptions of living-environment crises

**DOI:** 10.1186/s12889-021-10861-2

**Published:** 2021-04-22

**Authors:** Xiaoyun Li, Hongsheng Chen, Zhenjun Zhu

**Affiliations:** 1 College of City Construction, Jiangxi Normal University, Nanchang, 330022 China; 2 School of Architecture and Urban Planning, Shenzhen University, Shenzhen, 518060 Guangdong China; 3 College of Automobile and Traffic Engineering, Nanjing Forestry University, No.159 Longpan Road, Nanjing, 210037 China

**Keywords:** Life quality, Socioeconomic status, Crisis perception, Economic development, Developing countrie

## Abstract

**Background:**

Two common consequences of rapid economic development in developing countries are the improvement of residents’ quality of life but also environmental destruction. This study focuses on the relationship between residents’ perceptions on the life quality and their perception of environmental crises in China. The purpose of this study is to answer why people in developing countries have relatively high tolerance for environmental pollution caused by rapid economic development.

**Methods:**

Using ordered logistic regression models and the multi-level mixed effect ordinal regression model, and the data of the 2014 China Labour-force Dynamics Survey, 10,849 responses were analysed to evaluate public perceptions of living-environment crises. Specifically, perceptions relating to the following four living-environment crises were evaluated: (1) (un)employment; (2) public safety; (3) infectious disease; (4) pollution.

**Results:**

We found that respondents with higher levels of subjective well-being, social status, and sense of neighbourhood security expressed a lower level of concern for living-environment crises. The concern of urban residents was also significantly higher than that of rural residents for living-environment crisis. For rural respondents, neighbourhood population density was negatively correlated with the perception of employment, public safety, and infectious disease crises. For urban respondents, neighbourhood population density was positively correlated to the perception of public safety and pollution crises, and neighbourhood average income was positively correlated to the perception of infectious disease and pollution risks.

**Conclusions:**

Rapid economic development has greatly improved people’s well-being, but it has also produced many environmental pollution problems. To a certain extent, the improvement of the well-being will alleviate people’s worries about environmental pollution caused by the development of economy.

## Introduction

For developing countries, the relationships between economic development, the improvement of living standards, and environmental protection have always been an unbalanced triangle [1, 2]. Many studies have found that in developing countries, it is difficult to strike an effective balance between environmental protection and rapid economic development [2]. In the process of rapid economic development, the environment often loses out [3]; however, when economies are growing, countries and regions often adopt different means of protecting and restoring the environment, and environmental protection can itself produce positive economic and social benefits [4]. Although rapid economic development in developing countries may result in a certain degree of environmental damage, economic prosperity greatly improves residents’ quality of life. At the same time, improvements to life quality and social welfare because of rapid economic development can, to some extent, increase the tolerance of governments and residents to environmental damage [5]. For instance, Nicholas and Ilhan [6] found an inverted U-shaped association between CO_2_ emissions and income per capita for 14 Asian countries spanning the period 1990–2011. Indeed, developing countries seem to follow the development path of first developing their economy and then seeking appropriate environmental governance [7]. Therefore, when economic activity and urbanisation is promoted beyond a certain point, people’s tolerances of environmental pollution will be greatly reduced. For example, in Tangshan, an industrial city in China, Li and Tilt [8] found that residents gave environmental quality a relatively low priority but did rank it above economic concerns such as jobs and income.

In China, environmental pollution has become an increasing concern; as the country has grown to become the world’s second-largest economy, the Chinese government has heavily invested and implemented increasingly stringent environmental protection and restoration policies [7, 9]. Local governments are also becoming stricter in enforcing environmental protection policies; while large numbers of highly polluting activities have now been stopped across China [10, 11], striking the right balance between environmental protection and economic growth remains a major development issue. However, China’s emphasis on environmental governance has been continuously strengthened as the economy and society continue to develop. In the late 1970s, China implemented reform and opening-up policies and gradually completed the transition from a planned economy to a market economy [12, 13]. Driven by globalisation [14, 15], China’s economy has since achieved rapid growth through a series of institutional reforms and the full utilisation of labor resources, land resources, and mineral resources. At the same time, the institutional reform and the rapid development of the economy have greatly improved the quality of life of the Chinese population. Yet China’s rapid growth has been accompanied by increasing environmental damage [9, 16]. For example, air pollution has become a significant problem during the winter months; it has been shown that air pollution in China is directly related to the country’s energy and industrial structure [17, 18]. Furthermore, while the quality of urban living has been greatly improved, including the quality of healthcare, inequality between regions and between urban and rural areas has become more pronounced [19]. At the same time, many people’s lifestyles have not been rapidly altered by the processes of modernisation and urbanisation meaning that concepts such as environmental protection and sustainable development have not become popularised [20, 21].

Declines in the quality of the living environment in China are affected by a variety of factors including the widely implemented economic growth model, loose environmental protection policies, and weak uptake of sustainable development concepts. In academic discussions, there are many controversies about the relationships between economic development, environmental protection, and improvements to living standards. One view can be summed up as “economic prioritisation”, which emphasised economic development based on the exploitation of natural resources over inevitable environmental damage [22, 23]. This is supported by the belief that once economies are growing, the damaged environment can be repaired through industrial transformation and upgrading, such as the switch from resource-intensive to technology-intensive industries and the implementation of strict environmental protection policies [24]. Another view can be summarised as “environmental prioritarianism”, which favours strict environmental regulation on economic development, which will limit pollution and the associated negative impacts on quality of life; the underlying premise of “environmental prioritarianism” is that the benefits obtained through rapid economic development would be outweighed by associated damage to public health [25, 26].

Many existing researches has focused on the relationships between economic development, environmental protection, and social development at the macro level, while these relationships have rarely been considered at the level of individuals and communities. At the micro-level of economic development, environmental protection, and personal perception, some studies have demonstrated that the living environment has a significant impact on residents’ quality of life [27–29]; however, there has been little research on the impact of an individuals’ quality of life on their perception of their living environment. Evaluating these perception dynamics is crucial for understanding why—for many residents in rapidly developing countries—there is a higher tolerance for environmental pollution. To address this, we sought to evaluate the relationships between quality of life and the perception of environmental crises at the individual and community levels using detailed data for China obtained in 2014.

### Data and methods

#### Data

The 2014 China Labor-force Dynamics Survey conducted by the Centre for Social Science Survey of Sun Yat-sen University in China was used as the primary data source. This is a nationwide cross-sectional survey, using a multistage cluster stratified probability sampling procedure. The survey included detailed questionnaires on the socio-economic and well-being status of individual respondents and the characteristics of the community in which they lived. After removing the samples with missing information, a total of 10,849 samples were used to analyse Chinese residents’ perceptions of living-environment crises.

## Methods

Four kinds of living-environment crises (employment, public safety, infectious disease, and pollution) were selected for comparative research. The employment, public safety, infectious disease, and pollution crises perceptions of residents were used as the four dependent variables in the analysis. Of these, perceptions of the employment crisis represent respondents’ expectations for economic development. The public safety crisis reflects the social security situation of cities and neighbourhoods. Perceptions concerning infectious disease and pollution crises represent residents’ awareness of public health and environmental quality, respectively. Employment crisis perception was obtained by asking respondents “how likely do you think it is that you will be unemployed in the next five years?”; public safety crisis perception was gained by asking respondents “how likely do you think it is that you are to be victimised by a crime in the next five years?”; infectious disease crisis perception was evaluated by asking respondents “how likely do you think it is that you are to contract an infectious disease in the next five years?”; pollution crisis perception was assessed by asking respondents “how likely do you think it is that you will experience environmental pollution in the next five years?” In all cases, responses were given using a numerical scale with “1″ indicating the probability of the crisis is very small, and “4″ indicating the probability of the crisis is very large. We excluded respondents who chose the “don’t know/unsure” option. Since the dependent variable is ordinal, we used ordered logistic regression models and the multi-level mixed effect ordinal regression model to analyse the data.

The independent variables used in this study and their definitions are showed in Table 1. The specific variables included a respondent’s subjective well-being (3–15), quality of life compared with their neighbours (1–5), subjective social status (1–10), personal sense of neighbourhood security (1–4), social trust of their neighbours (1–5). Subjective well-being was evaluated by collating three interrelated variables, i.e., a respondent’s assessment of their own happiness (1–5), quality of life satisfaction (1–5), and family economic status satisfaction (1–5). Cronbach’s reliability coefficient was alpha = 0.83. The variables relating to community socio-economic status and environmental quality included neighbourhood population density, the average annual income of all registered community residents, the presence/absence of sources of pollution, the presence/absence of parks, the presence/absence of healthcare facilities (hospital or clinic), and the presence/absence of banking facilities. The control variables include annual personal income (yuan), the level of education (from 1 = never attend a school to 11 = holding a doctoral degree), gender, age, married status, job type, and neighbourhood type.

**Table 1:** The main independent variables used in the analysis and their definitions

Main variables	Variable definitions
**Neighbourhood-level variables**	
Neighbourhood population density	The ratio of the total permanent population of the community to the area of the community
Average annual income of registered community residents	Average annual income of registered community residents (those have local hukou)
The presence/absence of sources of pollution	Whether there is air pollution, soil pollution, water pollution, noise pollution, etc. within the administrative area of the community.
The presence/absence of parks	A square or park within the administrative area of the community.
The presence/absence of healthcare facilities	A hospital or private clinic within the administrative area of the community.
The presence/absence of banking facilities	A bank within the administrative area of the community.
**Individual-level variables**	
Subjective well-being	The subjective well-being score comes from the addition of three scores: self-evaluation of happiness in life, self-evaluation of satisfaction with life status, and self-evaluation of satisfaction with family economic status.
Quality of life compared to neighbours	Respondents’ evaluation of their living conditions based on comparison with their neighbours.
Subjective social status	Respondents used the MacArthur Scale of Subjective Social Status (social ladder) to evaluate their social position.
Personal sense of neighbourhood security	Respondents’ subjective evaluation of the social security of the community in which they live.
Social trust of neighbours	Respondents’ subjective evaluation of their social trust in neighbours in the community where they live.

In data analysis, we will first construct a regression model to analyze the impact of individual-level variables on residents’ perception of environmental crisis, and then construct a regression model to analyze the impact of neighborhood-level variables on residents’ perception of environmental crisis. In this way, we can quantitatively identify the relationship between residents’ quality of life and their perception of environmental crisis. In addition, due to there are significant differences in the living environment and lifestyles of residents between urban and rural neighborhoods in China, we will also compare the differences in the perception of environmental crises between urban residents and rural residents.

## Results

### Descriptive statistics

There are significant differences in the levels of economic development, environmental quality, and social welfare between urban and rural areas in China. Our results show that there are also significant differences in the perception of living-environmental crises (Table 2). Overall, rural residents perceived employment, public safety, infectious disease, and pollution crises to be less of an issue than urban residents. The main characteristics of the sampled group mainly include: subjective well-being (mean value = 10.68), personal sense of community security (mean value = 3.22) and neighbourhood social trust (mean value = 3.73) are relatively high, but the quality of life (mean value = 2.85) and subjective social status (mean value = 4.60) are relatively low (Table 3).

**Table 2:** Differences in the perception of living-environment crises between rural and urban respondents (*N* = 10,849)

	Rural residents (mean, S.D.)	Urban residents mean (mean, S.D.)	t-Test	*p*-value
Employment crisis perception (1–4)	1.41 (0.78)	1.61 (0.090)	−11.76	< 0.001
Public safety crisis perception (1–4)	1.16 (0.41)	1.27 (0.53)	−12.33	< 0.001
Infectious disease crisis perception (1–4)	1.21 (0.48)	1.41 (0.68)	−17.30	< 0.001
Pollution crisis perception (1–4)	1.47 (0.83)	1.81 (0.99)	−19.11	< 0.001

In each case, 1 = low risk perception, 4 = high risk perception

**Table 3:** Descriptive statistics of individual-level variables (*N* = 10,849)

Variables	Proportion, mean (SD)
Subjective well-being (3–15)	10.68 (2.42)
Quality of life compared to neighbours (1–5)	2.85 (0.64)
Subjective social status (1–10)	4.60 (1.66)
Personal sense of neighbourhood security (1–4)	3.22 (0.66)
Social trust of neighbours (1–5)	3.73 (0.84)
Annual personal income (yuan)	29,612.85 (75,635.64)
Education level (1–11)	3.60 (2.38)
Gender (%)	
Male	54.63
Female	45.37
Age (years old)	43.74 (12.85)
Married status (%)	
Single	8.91
Married	86.60
Divorced or widowed	4.49
Job type (%)	
Government department	9.02
Business employee	26.68
Self-employed and other	64.29
Neighbourhood type (%)	
Rural	67.33
Urban	32.67

### Relationships between perceptions of living-environment crises and quality of life

Table 4 shows the multilevel ordered logistic regression results for the living-environment crisis perception of residents. All of the three variables of well-being, subjective social status, and personal sense of neighbourhood security have a significant and negative effect on the four types of crisis perceptions, that is: for every additional unit of subjective well-being of the respondents, the perception of employment crisis, public safety crisis, infectious disease crisis and pollution crisis decrease by 0.087, 0.063, 0.054, 0.062 units respectively; for every additional unit of subjective social status of the respondents, the perception of employment crisis, public safety crisis, infectious disease crisis and pollution crisis decrease by 0.114, 0.078, 0.089, 0.098 units respectively; for every additional unit of subjective well-being of the respondents, the perception of employment crisis, public safety crisis, infectious disease crisis and pollution crisis decrease by 0.148, 0.321, 0.220, 0.336 units respectively. Subjective well-being and subjective social status have the greatest impact on respondents’ perceptions of (un) employment crisis, while personal sense of neighbourhood security has a greater impact on perceptions of pollution crisis and public safety crisis. The variable of quality of life compared with neighbours is only significantly and negatively related to respondents’ employment crisis perception. The variable of social trust of neighbours is negatively associated with respondents’ employment crisis perception and infectious disease crisis perception.

**Table 4:** Regression results on the relationships between perceptions of living-environment crises and quality of life

Main variables	Model 1: employment crisis		Model 2: public safety crisis		Model 3: infectious disease crisis		Model 4: pollution crisis	
	Coefficient	Robust S.E.	Coefficient	Robust S.E.	Coefficient	Robust S.E.	Coefficient	Robust S.E.
Subjective well-being	−0.087***	(0.013)	−0.063***	(0.017)	−0.054***	(0.015)	− 0.062***	(0.012)
Quality of life compared with neighbours	−0.120**	(0.049)	0.007	(0.053)	0.012	(0.049)	0.026	(0.046)
Subjective social status	−0.114***	(0.020)	−0.078***	(0.024)	−0.089***	(0.023)	−0.098***	(0.019)
Personal sense of neighbourhood security	−0.148***	(0.048)	−0.321***	(0.067)	−0.220***	(0.054)	−0.336***	(0.049)
Social trust of neighbours	−0.075*	(0.044)	−0.100	(0.061)	−0.118**	(0.049)	−0.046	(0.038)
**Control variables**								
(log) Annual personal income	−0.002	(0.011)	−0.003	(0.014)	0.001	(0.013)	0.008	(0.011)
Level of education	−0.025*	(0.014)	0.040**	(0.018)	0.089***	(0.018)	0.102***	(0.015)
Female (ref: male)	−0.199***	(0.051)	−0.092	(0.064)	−0.067	(0.050)	−0.134***	(0.044)
Age	−0.006**	(0.003)	−0.004	(0.003)	−0.003	(0.003)	−0.004*	(0.003)
Married status (ref: single)								
Married	−0.101	(0.087)	−0.127	(0.098)	−0.054	(0.093)	0.137	(0.095)
Divorced or widowed	−0.306*	(0.170)	− 0.221	(0.175)	−0.080	(0.181)	0.130	(0.153)
Job type (ref: government department)								
Business employee	0.705***	(0.106)	0.292***	(0.112)	0.178	(0.122)	0.140	(0.091)
Self-employed and other	0.288**	(0.124)	0.160	(0.120)	0.054	(0.118)	0.068	(0.097)
Urban neighbourhood (ref: rural neighbourhood)	0.470***	(0.113)	0.486***	(0.137)	0.521***	(0.131)	0.666***	(0.150)
cut1	−1.553***	(0.324)	−0.318	(0.398)	−0.085	(0.340)	−0.839***	(0.306)
cut2	−0.351	(0.327)	2.234***	(0.415)	1.958***	(0.350)	0.383	(0.308)
cut3	1.050***	(0.337)	4.172***	(0.442)	3.904***	(0.364)	2.064***	(0.314)
County-level variance	0.452***	(0.114)	0.378***	(0.124)	0.479***	(0.122)	0.540***	(0.169)
Neighbourhood-level variance	0.386***	(0.082)	0.689***	(0.133)	0.589***	(0.122)	0.997***	(0.193)
Number of counties	209		209		209		209	
Number of neighbourhoods	401		401		401		401	
Number of individuals	10,849		10,849		10,849		10,849	
Log likelihood	− 8944.318		− 5168.108		− 6402.683		− 9380.726	
Chi-squared	396.477		145.051		200.957		255.080	

Standard errors shown in parentheses. * *p* < 0.10, ** *p* < 0.05, *** *p* < 0.01

From the above results, we can see that respondents with higher socioeconomic status were more likely to believe that they will not suffer from living-environment crisis in the future. This is mainly related to social capital; people of higher socioeconomic status tend to have more social capital (i.e., personal ability, economic ability, social connections, health, and political power) [53]. Those with higher socioeconomic status are more optimistic about economic development, and they are more resilient to economic crises than those with lower economic and social status. And they can also afford to live far away from pollution and areas with poor sanitation, and can take more defensive measures against health crises by seeking more timely treatment in the event of a pandemic.

The level of education has a negative impact on the respondents’ perception of employment crisis, while it has a positive impact on the other three crisis perceptions. The former may be because people with a high level of education have stronger professional abilities and are less likely to lose their jobs, while the latter may be because education can raise people’s awareness of the crisis in the living environment. Women and older respondents considered the risk of unemployment and pollution to be significantly lower than men and younger respondents, respectively. In China, professions are directly related to class, social welfare, etc. Among them, civil servants (work in government department) are one of the most respected professions. There are also significant differences in people’s perception of living-environment crisis in different occupations. Respondents who worked for companies perceived a significantly higher employment crisis and public safety crisis than those who worked in government. And respondents who were self-employed perceived a significantly higher employment crisis than those who worked in government. In China, the difference between urban and rural living environment is very large, and the living environment of urban neighbourhood is generally better than that of rural neighbourhood. In terms of the differences in perceptions of living environment crisis perceptions among urban and rural residents, we find that respondents living in urban neighbourhood had a significantly higher perception of all four types of crisis than rural residents.

### Relationships between perceptions of living-environment crises and neighbourhood characteristics

Table 5 shows the multilevel order logistic regression results for rural respondents. For neighbourhood-level variables, the neighbourhood population density has a slight impact on the respondents’ perception of the crisis of living environment. Respondents living near the pollution source were more likely to express a higher level of concern for employment, public safety, infectious disease, and pollution crises than those living far away from pollution sources. Pollution sources appeared to have the most significant impact on the perceptions of rural residents towards living-environment crises. For respondents living in urban neighbourhood (Table 6), a higher neighbourhood population density was associated with a stronger perception of public safety and pollution crises, and those living in communities with a higher average annual income were more likely to express more concern for infectious disease and pollution crisis. In terms of the impact of public services, people living in communities with hospitals or clinics were showed less concern for unemployment and public safety crises; and those living in communities with banks were less concerned about employment crises. It can be seen from the above results that the social structure and public service facilities of communities have a certain impact on people’s perception of environmental crisis.

**Table 5:** Regression results on the relationships between rural respondents’ perceptions of living-environment crises and neighbourhood characteristics

Main variables	Model 5: employment crisis		Model 6: public safety crisis		Model 7: infectious disease crisis		Model 8: pollution crisis	
	Coefficient	Robust S.E.	Coefficient	Robust S.E.	Coefficient	Robust S.E.	Coefficient	Robust S.E.
Neighbourhood population density	− 0.005***	(0.001)	− 0.004***	(0.001)	−0.004*	(0.002)	0.001*	(0.000)
Average annual income of registered community residents	0.068	(0.144)	0.088	(0.148)	0.039	(0.166)	0.071	(0.147)
The presence/absence of sources of pollution (ref: yes)	−0.372**	(0.152)	−0.556**	(0.224)	−0.419*	(0.219)	−1.219***	(0.311)
The presence/absence of parks (ref: no)	0.250	(0.198)	0.088	(0.215)	0.194	(0.216)	0.416*	(0.227)
The presence/absence of healthcare facilities (ref: no)	0.128	(0.330)	0.030	(0.358)	0.470	(0.351)	0.547	(0.361)
The presence/absence of banking facilities (ref: no)	0.316	(0.194)	0.466*	(0.250)	0.201	(0.239)	−0.318	(0.287)
**Individual-level variables**								
Subjective well-being	−0.093***	(0.018)	−0.074***	(0.024)	−0.046**	(0.022)	−0.035*	(0.020)
Quality of life compared to neighbours	−0.048	(0.068)	0.092	(0.074)	−0.015	(0.067)	−0.010	(0.056)
Subjective social status	−0.085***	(0.028)	−0.052	(0.032)	−0.019	(0.030)	−0.075***	(0.029)
Personal sense of neighbourhood security	−0.129	(0.082)	−0.376***	(0.121)	−0.250***	(0.088)	−0.326***	(0.068)
Social trust of neighbours	−0.035	(0.069)	−0.025	(0.104)	−0.102	(0.082)	−0.032	(0.058)
(log) Annual personal income	0.023	(0.017)	0.014	(0.019)	0.014	(0.021)	0.005	(0.014)
Level of education	−0.034	(0.032)	0.029	(0.037)	0.089**	(0.037)	0.128***	(0.030)
Female (ref: male)	−0.244***	(0.089)	0.041	(0.100)	0.065	(0.082)	−0.146**	(0.074)
Age	−0.005	(0.004)	0.000	(0.004)	0.001	(0.004)	−0.002	(0.004)
Married status (ref: single)								
Married	−0.063	(0.140)	−0.384**	(0.174)	−0.249	(0.160)	0.095	(0.149)
Divorced or widowed	0.016	(0.260)	−0.391	(0.322)	−0.265	(0.290)	0.161	(0.244)
Job type (ref: government department)								
Business employee	0.339	(0.248)	−0.154	(0.305)	0.434	(0.295)	0.190	(0.217)
Self-employed and other	− 0.257	(0.243)	−0.222	(0.297)	0.317	(0.291)	0.150	(0.235)
cut1	−1.218*	(0.675)	−0.344	(0.790)	0.868	(0.676)	−0.622	(0.703)
cut2	−0.084	(0.694)	2.272***	(0.829)	3.091***	(0.699)	0.613	(0.709)
cut3	1.343*	(0.696)	4.696***	(0.916)	5.320***	(0.700)	2.425***	(0.725)
County-level variance	0.564***	(0.213)	0.314	(0.217)	0.668***	(0.228)	0.520	(0.448)
Neighbourhood-level variance	0.377**	(0.180)	0.981***	(0.261)	0.672***	(0.181)	1.388***	(0.434)
Number of counties	111		111		111		111	
Number of neighbourhoods	168		168		168		168	
Number of individuals	5369		5369		5369		5369	
Log likelihood	− 4013.412		− 2167.552		− 2650.853		− 4104.908	
Chi-squared	233.215		60.361		56.559		144.296	

Standard errors shown in parentheses. * *p* < 0.10, ** *p* < 0.05, *** *p* < 0.01

**Table 6:** Regression results on the relationships between urban respondents’ perceptions of living-environment crises and neighbourhood characteristics

Main variables	Model 9: employment crisis		Model 10: public safety crisis		Model 11: infectious disease crisis		Model 12: pollution crisis	
	Coefficient	Robust S.E.	Coefficient	Robust S.E.	Coefficient	Robust S.E.	Coefficient	Robust S.E.
Neighbourhood population density	0.002	(0.002)	0.005***	(0.002)	0.003	(0.003)	0.005*	(0.003)
Average annual income of registered community residents	0.016	(0.013)	0.014	(0.016)	0.026*	(0.014)	0.036**	(0.015)
The presence/absence of sources of pollution (ref: yes)	−0.117	(0.142)	− 0.067	(0.210)	− 0.215	(0.182)	− 0.274	(0.193)
The presence/absence of parks (ref: no)	0.126	(0.134)	−0.170	(0.194)	−0.235	(0.167)	0.076	(0.151)
The presence/absence of healthcare facilities (ref: no)	−0.364*	(0.191)	−0.390*	(0.215)	0.011	(0.209)	−0.277	(0.169)
The presence/absence of banking facilities (ref: no)	−0.238*	(0.141)	−0.109	(0.182)	0.114	(0.167)	0.089	(0.161)
**Individual-level variables**								
Subjective well-being	−0.125***	(0.023)	−0.065**	(0.030)	−0.063**	(0.026)	−0.087***	(0.021)
Quality of life compared to neighbours	−0.302***	(0.082)	−0.140	(0.102)	−0.048	(0.104)	0.007	(0.097)
Subjective social status	−0.134***	(0.035)	−0.084*	(0.044)	−0.148***	(0.041)	−0.155***	(0.032)
Personal sense of neighbourhood security	−0.149*	(0.079)	−0.225**	(0.103)	−0.131*	(0.078)	−0.376***	(0.080)
Social trust of neighbours	−0.017	(0.057)	0.000	(0.081)	−0.033	(0.068)	0.008	(0.056)
(log) Annual personal income	−0.033	(0.021)	−0.002	(0.027)	0.022	(0.023)	0.017	(0.024)
Level of education	−0.034*	(0.018)	0.011	(0.021)	0.060***	(0.021)	0.092***	(0.020)
Female (ref: male)	−0.080	(0.086)	−0.116	(0.114)	−0.075	(0.085)	−0.064	(0.078)
Age	−0.003	(0.006)	−0.009	(0.006)	−0.012**	(0.005)	−0.002	(0.005)
Married status (ref: single)								
Married	−0.062	(0.143)	−0.026	(0.175)	0.130	(0.157)	0.156	(0.160)
Divorced or widowed	−0.373	(0.274)	0.030	(0.268)	0.062	(0.264)	0.188	(0.242)
Job type (ref: government department)								
Business employee	0.604***	(0.152)	0.330**	(0.141)	0.167	(0.155)	0.013	(0.119)
Self-employed and other	0.407**	(0.180)	0.224	(0.150)	0.008	(0.148)	−0.005	(0.128)
cut1	−3.664***	(0.481)	−1.459***	(0.563)	−0.981*	(0.526)	−2.246***	(0.524)
cut2	−2.411***	(0.483)	0.953	(0.605)	0.821	(0.525)	−1.059**	(0.517)
cut3	−1.102**	(0.484)	2.596***	(0.643)	2.594***	(0.568)	0.615	(0.517)
County-level variance	0.077	(0.132)	0.110	(0.178)	0.000	(0.000)	0.330**	(0.131)
Neighbourhood-level variance	0.263*	(0.144)	0.478**	(0.238)	0.515***	(0.106)	0.249**	(0.119)
Number of counties	93		93		93		93	
Number of neighbourhoods	122		122		122		122	
Number of individuals	2576		2576		2576		2576	
Log likelihood	− 2580.546		− 1651.941		− 2106.719		− 2864.317	
Chi-squared	273.034		108.072		68.233		172.655	

Standard errors shown in parentheses. * *p* < 0.10, ** *p* < 0.05, *** *p* < 0.01

## Discussion

With the rapid growth of its economy, the living environment in China has been seriously damaged [30, 31]. However, in the absence of direct harm to the well-being of the individual, different groups have different perceptions of the environmental crisis [32–34]. Our principal finding is that there is a negative correlation between subjective well-being/ social status and the perceptions of living-environment crises. The results suggest that subjective well-being reduces anxiety about environmental crisis. This also demonstrates people’s tolerance for the environmental damage caused by economic growth at the individual level. When the economy is booming, social mobility is accelerated and quality of life will improve [35, 36] which reduces anxiety about environmental crises. Furthermore, people who express a higher level of social status typically have access to more social resources and the ability to choose their living environment, which further reduces their sense of living environment crisis [37, 38].

This research tries to better understand the relationship between “happy life” and perception of crisis in the living environment, arising from economic development and rapid urbanization in developing countries. But we admit that this research has certain shortcomings. First, the perception of the living environment crisis is closely related to the individual’s education level and the ability to obtain relevant information [39, 40]. Second, the relationship between subjective well-being and perception of living environment crisis may be an unstable relationship, because people’s subjective well-being will be affected by multiple factors and personal experience [36, 41, 42], and people’s perception of living environment crisis will change with time.

For policy, first, more effort is required to find a development path that enables economic prosperity alongside ecological protection, and which improves people’s life quality and socioeconomic status while minimising environmental damage. Second, the awareness of residents to environmental crises should be improved. Education is one of the most effective ways to achieve this, especially in developing countries where poor education represents one of the most important restrictions on environmental protection. Third, changes are required in our daily way of life and approach to production. We emphasise the need for healthier and more environmentally friendly ways of living and production. Due to limited data, we need to conduct further research on some unanswered questions in the future. For example, although this study emphasizes the impact of the community environment on residents’ perception of environmental crisis, it is still unclear to what extent the community environment’s impact on residents’ perception of environmental crisis. And we try to use the results of this research to explain people’s tolerance for environmental crises caused by economic development, but we need to further analyze the relationship between economic development, people’s life quality and perceptions of environmental crises. Furthermore, because this research uses a secondary data, which does not have objective environmental quality evaluation data about the respondents’ residence, so respondents’ subjective perceptions of environmental crises may deviate from the actual environmental quality.

## Conclusions

Focusing on four types of living-environment crises in China, we found that residents with a higher level of subjective well-being, social status, and sense of neighbourhood security are less aware of employment, public safety, infectious disease, and pollution crises. Educational level was negatively associated with respondents’ perception of the employment crisis but was positively associated with concern about public safety, infectious disease, and pollution. Urban residents expressed a significantly higher level of concern about living-environment crises than rural residents.

## Data Availability

The data description and data application used in this study can be found in the website: http://ssa.sysu.edu.cn/article/1994.
